# Distinct role of histone chaperone Asf1a and Asf1b during fertilization and pre-implantation embryonic development in mice

**DOI:** 10.1186/s13072-021-00430-7

**Published:** 2021-12-14

**Authors:** Xuemei Wang, Lu Wang, Jie Dou, Tianjiao Yu, Pengbo Cao, Na Fan, Uyunbilig Borjigin, Buhe Nashun

**Affiliations:** State Key Laboratory of Reproductive Regulation and Breeding of Grassland Livestock, School of Life Sciences, Inner Mongolia University, 24 Zhaojun Road, Yuquan District, Hohhot, 010070 Inner Mongolia China

**Keywords:** Asf1a, Asf1b, Pre-implantation embryos, H3K56ac, Oct4, PCNA

## Abstract

**Background:**

Asf1 is a well-conserved histone chaperone that regulates multiple cellular processes in different species. Two paralogous genes, Asf1a and Asf1b exist in mammals, but their role during fertilization and early embryogenesis remains to be investigated further.

**Methods:**

We analyzed the dynamics of histone chaperone Asf1a and Asf1b in oocytes and pre-implantation embryos in mice by immunofluorescence and real-time quantitative PCR, and further investigated the role of Asf1a and Asf1b during fertilization and pre-implantation development by specific Morpholino oligos-mediated knock down approach.

**Results:**

Immunofluorescence with specific antibodies revealed that both Asf1a and Asf1b were deposited in the nuclei of fully grown oocytes, accumulated abundantly in zygote and 2-cell embryonic nuclei, but turned low at 4-cell stage embryos. In contrast to the weak but definite nuclear deposition of Asf1a, Asf1b disappeared from embryonic nuclei at morula and blastocyst stages. The knockdown of Asf1a and Asf1b by specific Morpholino oligos revealed that Asf1a but not Asf1b was required for the histone H3.3 assembly in paternal pronucleus. However, knockdown of either Asf1a or Asf1b expression decreased developmental potential of pre-implantation embryos. Furthermore, while Asf1a KD severely reduced H3K56 acetylation level and the expression of Oct4 in blastocyst stage embryos, Asf1b KD almost eliminated nuclear accumulation of proliferating cell marker-PCNA in morula stage embryos. These results suggested that histone chaperone Asf1a and Asf1b play distinct roles during fertilization and pre-implantation development in mice.

**Conclusions:**

Our data suggested that both Asf1a and Asf1b are required for pre-implantation embryonic development. Asf1a regulates H3K56ac levels and Oct4 expression, while Asf1b safeguards pre-implantation embryo development by regulating cell proliferation. We also showed that Asf1a, but not Asf1b, was necessary for the assembly of histone H3.3 in paternal pronuclei after fertilization.

**Supplementary Information:**

The online version contains supplementary material available at 10.1186/s13072-021-00430-7.

## Introduction

The basic structural unit of chromatin is the nucleosome, which consists of 146 bp DNA wrapped around a histone octamer containing two molecules each of histones H2A, H2B, H3, and H4 [1]. Assembly and disassembly of nucleosomes occur during DNA related processes, such as DNA replication, DNA repair, transcription and recombination [2]. The basic steps for nucleosome assembly involve deposition of the H3–H4 dimers onto the DNA, and then combining the two histone H2A–H2B dimers to form a complete nucleosome core particle [3]. Histone chaperones, as the special class of proteins with high affinity for histone binding, play a key role in nucleosome dynamics [2].

Asf1 is a histone H3–H4 chaperone that is well conserved in eukaryotes [4, 5]. The histone chaperone Asf1 participates not only in CAF-1-mediated DNA replication-dependent nucleosome assembly [6–9], but also in Hira-mediated DNA replication-independent nucleosome assembly [9, 10]. Additionally, Asf1 is also involved in regulation of epigenetic modifications in different species. ASF1 is required for acetylation of H3K56 in yeast, *Drosophila* and human cells [11–13] and studies in yeast and human cells suggested that ASF1 and H3K56ac act within the same pathway under certain conditions [13].

In most vertebrates, there are two distinct Asf1 paralogous genes, Asf1a and Asf1b [4, 14, 15]. In human cells, they are 71% homologous, distinguished mainly by C-terminal sequences [4]. ASF1a is mainly involved in DNA repair and cellular senescence in human cells [15] and the absence of Asf1a leads to cell death in vertebrate cells [16]. Moreover, mutations in the Asf1a gene lead to embryonic lethality at midgestation in mice [17]. In contrast, Asf1b is dispensable for mouse development. However, its expression is developmentally regulated in the germ cells of both sexes and required for meiotic entry [18]. Additionally, Asf1b is highly expressed in multiple types of cancers including cervical cancer and prostate cancer [19, 20] and mainly involved in cell proliferation [21].

In mammals, the sperm genome is packaged by protamine into a highly condensed chromatin. Soon after fertilization, the sperm is gradually de-condensed and protamine is replaced by maternal histone to form the male pronucleus [22, 23]. Histone H3.3 has been shown to exclusively incorporate into the paternal genome immediately after fertilization [24] and requires coordination of histone chaperone Hira to ensure proper assembly of paternal nucleosome. Recent reports suggested that histone chaperone Asf1 is also involved in the paternal nucleosome assembly, since depletion of the maternal pool of Asf1 in *Drosophila* oocytes leads to failure in decondensation of the male pronucleus after fertilization [25]. However, the role of Asf1a and Asf1b during fertilization and embryogenesis in mammalian species remains largely unknown.

In this study, we characterized dynamic changes in nuclear accumulation of histone chaperone Asf1a and Asf1b in mouse oocytes and early embryos. Moreover, taking advantage of Morpholino oligos to silence the expression of Asf1a or Asf1b, we showed that both Asf1a and Asf1b were required for pre-implantation embryonic development. Furthermore, while Asf1a is required for the deposition of histone H3.3 in the paternal genome after fertilization and regulates embryonic H3K56ac and Oct4 level, Asf1b mainly regulates cell proliferation during early embryogenesis.

## Results

### The nuclear dynamics of Asf1a and Asf1b in mouse oocytes and pre-implantation embryos

**Fig. 1:**
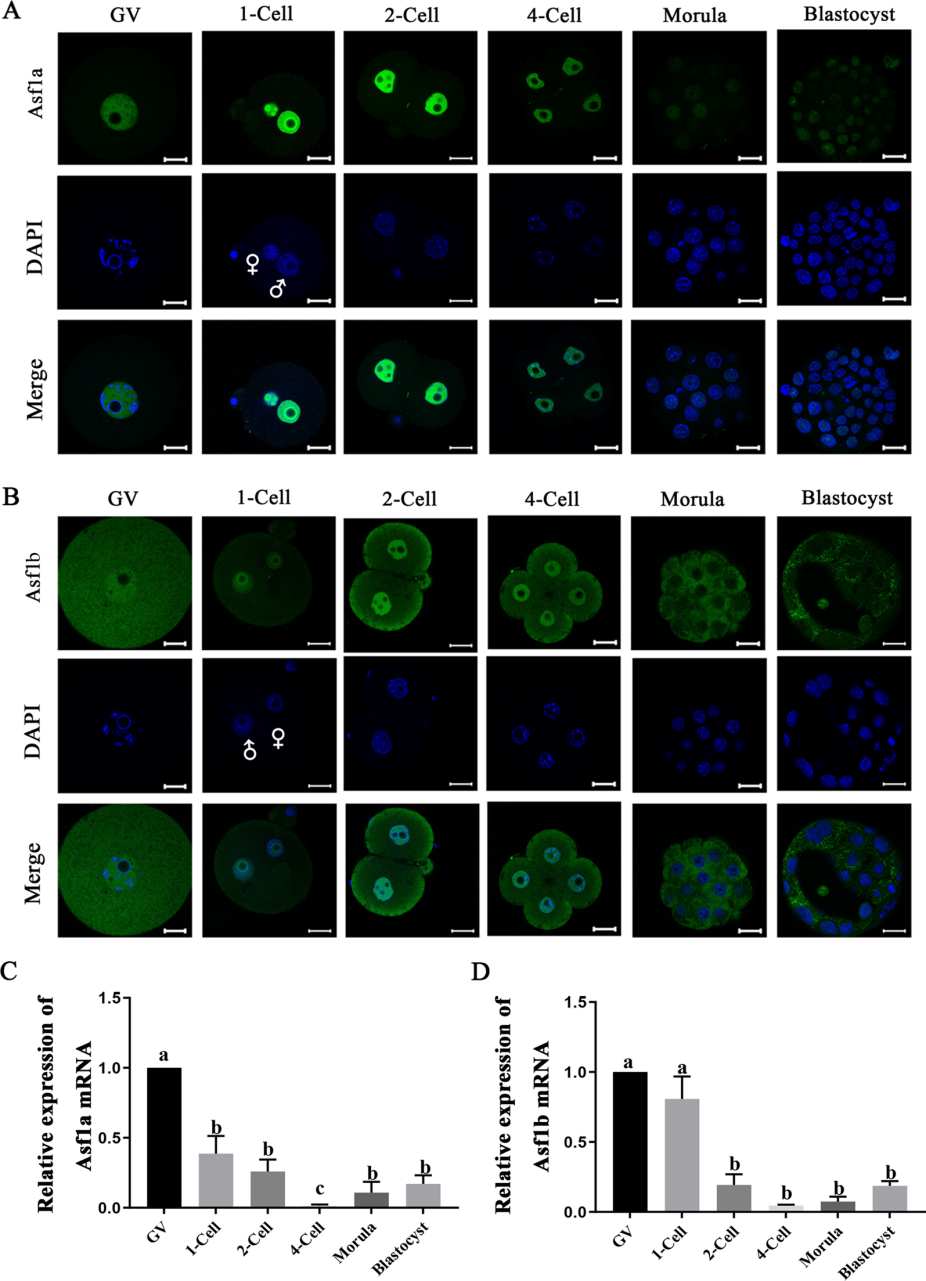
Dynamic changes of the nuclear deposition of Asf1a and Asf1b in mouse oocytes and pre-implantation embryos. **A**, **B** Immunofluorescence staining for Asf1a (green) and Asf1b (green) in mouse oocytes at the germinal vesicle (GV), and pre-implantation embryos at the one-cell (1-cell), two-cell (2-cell), four-cell (4-cell), morula (morula) and blastocyst (blastocyst) stages. 9 to 22 embryos were analyzed in each group. DNA was stained with DAPI (blue). Scale bar, 20 µm. **C**, **D** Relative expression of histone Asf1a mRNA (**C**) and Asf1b mRNA (**D**) in mouse GV oocytes and pre-implantation embryos. The mean value for GV oocytes was set as 1, and relative values for all other samples were calculated accordingly. Data were obtained from three independent experiments and presented as mean ± SEM. Significant difference (a versus b, b versus c; *P* < 0.05) was determined by the one-way ANOVA and Student’s *t*-test

We first examined the dynamics of the histone chaperone Asf1a and Asf1b in oocytes and pre-implantation embryos using specific antibodies (Additional file 1: Fig. S1). Asf1a was readily detected in the GVs of fully grown oocytes. After fertilization, Asf1a was present in both male and female pronuclei, and the fluorescent signal became higher than that observed in GV-stage oocytes. At the 2-cell stage, the signal intensity remained higher than that in GV-stage oocytes. However, it decreased at the 4-cell stage and further decreased at the morula and blastocyst stages (Fig. 1A and Additional file 1: Fig. S2A). Similarly, nuclear localization of the histone chaperone Asf1b was readily detected in GV-stage oocytes. The signal remained intense in 1-cell and 2-cell stage embryos and decreased at the 4-cell stage (Fig. 1B and Additional file 1: Fig. S2B). Remarkably, nuclear deposition of Asf1b was not detected either in the morula or in the blastocyst stage embryos (Fig. 1B), which was in stark contrast to Asf1a, whose nuclear localization was weak but clearly detected in the morula and blastocyst stage embryos (Fig. 1A).We then analyzed expression dynamics of mRNA for Asf1a and Asf1b in oocytes and pre-implantation embryos by qPCR. Consistent with previous RNA-seq analysis [26] (Additional file 1:Fig. S3), both Asf1a and Asf1b showed very similar mRNA expression pattern. Compared to the GV-stage oocytes, the expression level continuously decreased after fertilization and reached the lowest level at the 4-cell stage, followed by gradual increase in the morula and blastocyst stage embryos (Fig. 1C, D). Taken together, these results suggested that both histone chaperone Asf1a and Asf1b were abundantly expressed in oocytes prior to fertilization and localized in the nucleus of early cleavage stage embryos, indicating their possible involvement during fertilization and early embryogenesis.

### Asf1a but not Asf1b was required for the histone H3.3 deposition in the male pronucleus after fertilization

**Fig. 2:**
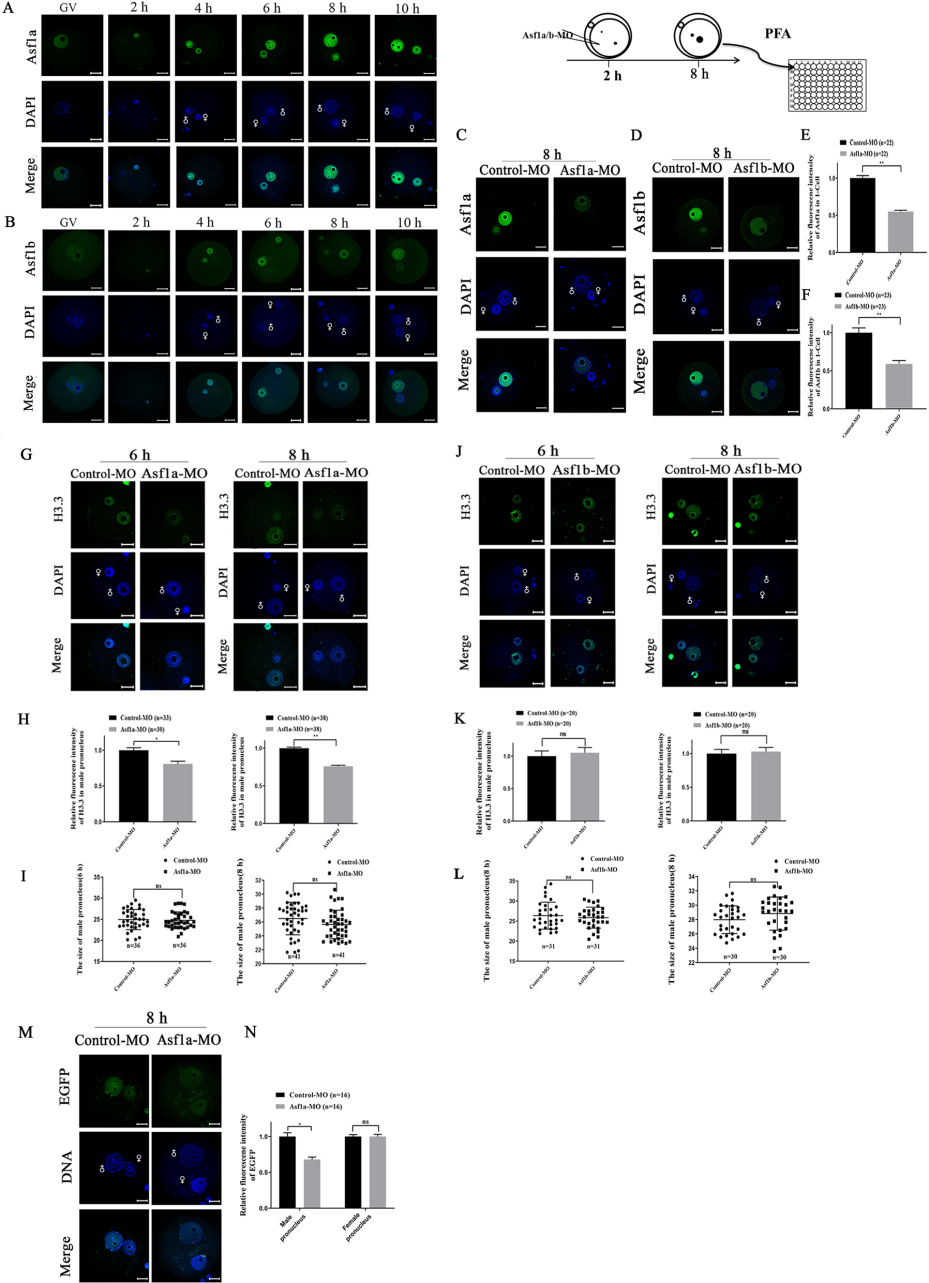
Asf1a, but not Asf1b, was required for histone H3.3 assembly in paternal genome. **A**, **B** Nuclear localization of Asf1a and Asf1b were examined by immunofluorescence staining in zygotes at 2, 4, 6, 8 and 10 hpi, respectively. 11–36 embryos were analyzed in each group. **C**, **D** Representative images of zygotes stained with antibodies against Asf1a and Asf1b. Asf1a-MO, Asf1b-MO or Control-MO was microinjected in zygotes at 2 hpi and the knockdown efficiency was examined in zygote at 8 hpi by immunofluorescence staining. **E**, **F** Quantification of the fluorescence intensities of Asf1a and Asf1b in zygotes. **G**, **J** Representative images of zygotes stained with antibodies against histone H3.3. Asf1a-MO, Asf1b-MO or Control-MO was microinjected into zygote at 2 hpi and immunostained with H3.3 antibody at 6 and 8 hpi, respectively. **H**, **K** Quantification of H3.3 fluorescence intensity in the Asf1a KD and Asf1b KD zygotes at 6 hpi and 8 hpi, respectively. **I**, **L** Quantification of the size of the paternal pronuclei in zygotes. Each dot represents a paternal pronucleus examined. Control-MO, Asf1a-MO or Asf1b-MO was microinjected in zygote at 2 hpi and the diameter of the paternal pronuclei was measured at 6 hpi and 8 hpi, respectively. Three horizontal lines from top to bottom represent the upper quartile, median and lower quartile, respectively. **M** Representative images of zygotes stained with antibody against EGFP. MII oocytes were collected from  H3.3B-EGFP knock-in mice and fertilized in vitro. Asf1a-MO or Control-MO was microinjected into zygote at 2 hpi and immunostained with EGFP antibody at 8 hpi. **N** Quantification of the EGFP fluorescence intensity in the male and female pronuclei. Data were presented as mean ± SEM, and analyzed by Student’s t-test. N represents the number of embryos or blastomeres examined

After fertilization, de novo nucleosome assembly in the male pronucleus is a crucial step toward successful genomic reprogramming. In *Drosophila,* Asf1 handovers H3.3–H4 dimers to the HIRA complex prior to histone deposition on paternal genome and is required for formation of the male pronucleus [25]. Interestingly, Asf1 does not reside in the decondensing sperm nucleus *in Drosophila* embryo [25]. In order to examine if it is also the case in mammalian species, we first examined localization dynamics of histone chaperone Asf1a and Asf1b by immunofluorescence staining in zygotes at different pronuclear stages. Both Asf1a and Asf1b were clearly detected from the maternal and paternal pronuclei in zygotes at 2, 4, 6, 8, and 10 hpi (hour post insemination) and the signal became intense in the later stage zygotes (Fig. 2A, B), indicating that mammalian Asf1 may function differently from its counterpart in *Drosophila*.

Since Morpholino oligos (MO) provides better specificity, low toxicity and long-time efficiency [27–29], we used specific Morpholino oligos to down-regulate the expression of histone chaperone Asf1a and Asf1b in zygotes, aiming to further dissect their roles in nucleosome assembly and paternal pronucleus formation. The Morpholino against Asf1a (Asf1a-MO), Asf1b (Asf1b-MO) and control Morpholino (Control-MO) was microinjected individually into zygotes at 2 hpi and the KD efficiency was examined in zygotes at 8 hpi (Fig. 2C, D). Compared to the Control-MO injected zygotes, nuclear accumulation of Asf1a and Asf1b was reduced more than 40% in Asf1a-MO or Asf1b-MO injected zygotes (Fig. 2E, F), demonstrating the effective inhibitory effect of translation. Since maternal H3.3 incorporates preferentially into the male pronucleus after fertilization [24], we set out to examine if nuclear accumulation of H3.3 was affected by the Asf1a knockdown using specific H3.3 antibody [30, 31] staining (Additional file 1: Fig. S5). Compared to the control, Asf1a-MO significantly reduced H3.3 accumulation in the male pronucleus and the fluorescence intensity of H3.3 was dropped by 20% at 6 hpi and 24% at 8 hpi, respectively (Fig. 2G, H). This finding was further confirmed using zygotes derived from H3.3B-EGFP knock-in mice [32]. MII oocytes were collected from H3.3B-EGFP female mice and fertilized in vitro. Then, the zygotes were microinjected with Asf1a-MO at 2 hpi and examined by EGFP antibody staining at 8 hpi. As expected, Asf1a knockdown significantly reduced EGFP-H3.3 deposition in the paternal but not in the maternal pronuclei (Fig. 2M, N). In contrast, Asf1b knockdown did not affect accumulation of H3.3 in paternal or maternal pronuclei (Fig. 2J, K). We continued to measure the diameter of paternal pronuclei and found that neither Asf1a nor Asf1b knockdown affected the size of the male pronucleus (Fig. 2I, L). Taken together, these results suggested that Asf1a but not Asf1b is required for histone H3.3 loading in the paternal genome after fertilization and sperm decondensation happens independently of the histone chaperones Asf1a and Asf1b.

### Knockdown of Asf1a or Asf1b had a detrimental effect on pre-implantation embryonic development

**Fig. 3:**
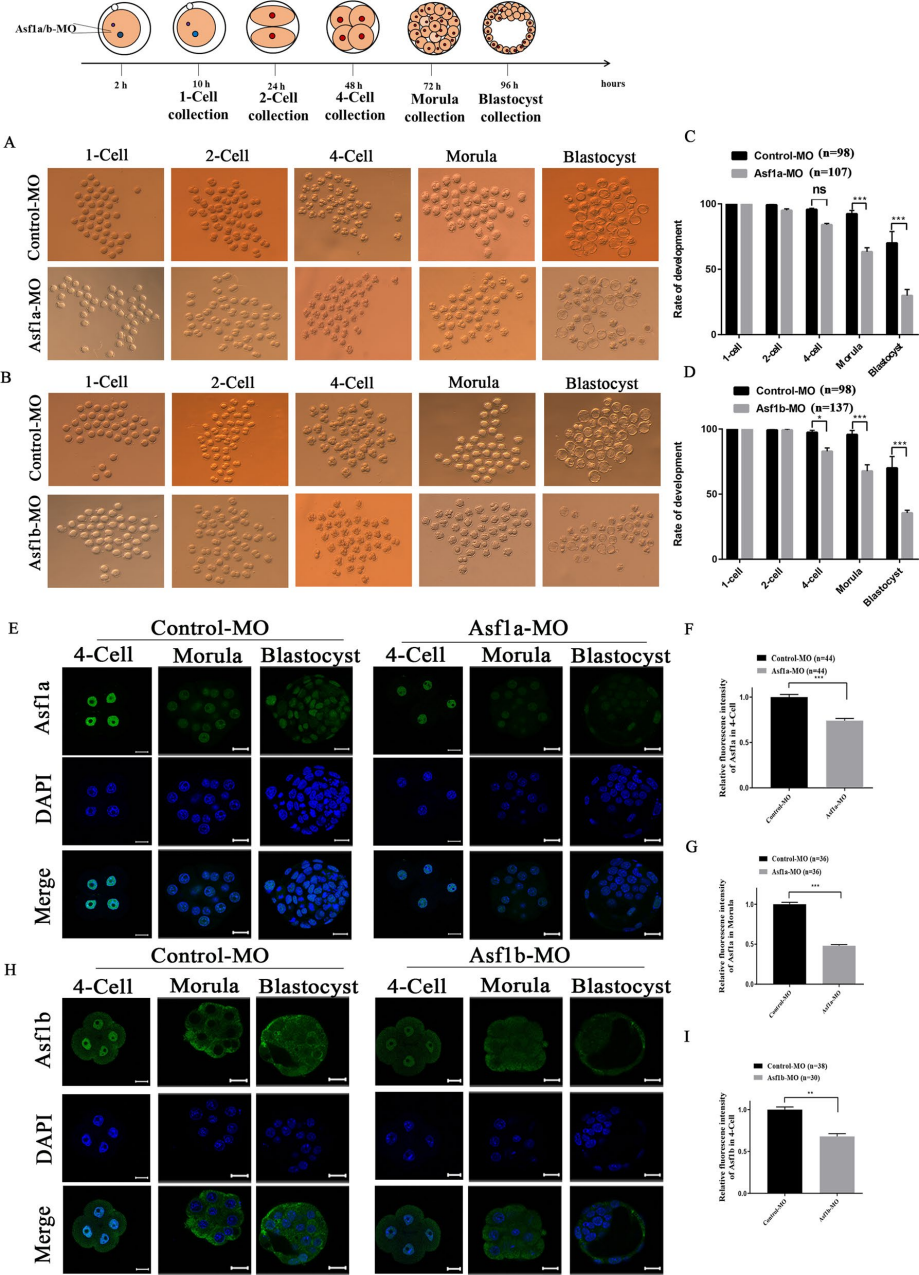
Knockdown of Asf1a or Asf1b had a detrimental effect on developmental progression. **A**, **B** Representative images of embryos developed from Control-MO, Asf1a-MO, or Asf1b-MO microinjected zygotes. **C**, **D** Quantification of developmental progression. Data were obtained from three independent experiments and the total number of embryos used for the quantification was as indicated. (Control-MO, *n* = 98; Asf1a-MO, *n* = 107; Asf1b-MO, *n* = 137). **E** Immunofluorescence detection of Asf1a in 4-cell, morula and blastocyst stage embryos developed from Control-MO (left) or Asf1a-MO (right) microinjected zygotes. **F**, **G** Quantification of Asf1a fluorescence intensity in 4-cell (Control-MO, *n* = 44; Asf1a-MO, *n* = 44) and morula (Control-MO, *n* = 36; Asf1a-MO, *n* = 36). **H** Immunofluorescence detection of Asf1b in 4-cell, morula and blastocyst stage embryos developed from Control-MO (left) or Asf1b-MO (right) microinjected zygotes. **I** Quantification of Asf1b fluorescence intensity in 4-cell stage embryos. (Control-MO, *n* = 38; Asf1b-MO, *n* = 30). All quantification data were presented as mean ± SEM, and analyzed using Student’s *t*-test, *P < 0.05, **P < 0.01 and ***P < 0.001; DNA was stained with DAPI, Scale bar 20 µm

To investigate the effect of Asf1a or Asf1b knockdown on pre-implantation development, zygotes (2 hpi) were microinjected with specific MO against Asf1a or Asf1b, respectively, and examined their development rates. In agreement with previous studies [33], knockdown of Asf1a did not affect developmental rate of embryos at 2-cell and 4-cell stages, but significantly decreased that of the morula and blastocyst stage embryos (Fig. 3A, C). In contrast, the knockdown of Asf1b significantly reduced developmental rates of pre-implantation embryos apart from 2-cell stage (Fig. 3B, D). These results suggested that Asf1a and Asf1b are required for pre-implantation development.

In order to verify that the decreased developmental potential was due to the knockdown effect of the two histone chaperones, we examined nuclear accumulation of Asf1a and Asf1b in 4-cell, morula and blastocyst stage embryos (Fig. 3E, H). Immunofluorescence staining by specific antibody revealed that translational inhibitory effect of the Asf1a-MO persisted till blastocyst stage (Fig. 3E and Additional file 1: Fig. S4A) and quantification of the fluorescence signal confirmed that nuclear accumulation of Asf1a in 4-cell and morula were significantly reduced (Fig. 3F, G). Since Asf1b disappears from embryonic nuclei from morula stage onward (Figs. 1B, 3H), it was only possible to examine the knockdown effect of Asf1b-MO in 4-cell stage embryos. Similarly, microinjection of Asf1b-MO in zygotes significantly reduced Asf1b accumulation in 4-cell embryonic nuclei (Fig. 3H, I). Collectively, these results demonstrated that microinjection of specific MO in zygote effectively inhibited translation of Asf1a or Asf1b during entire pre-implantation development and provided support for the hypothesis that both Asf1a and Asf1b were required for the early embryonic development in mice.

### Asf1a was required for maintenance of H3K56ac, while Asf1b was mainly involved in cell proliferation in early embryos

**Fig. 4:**
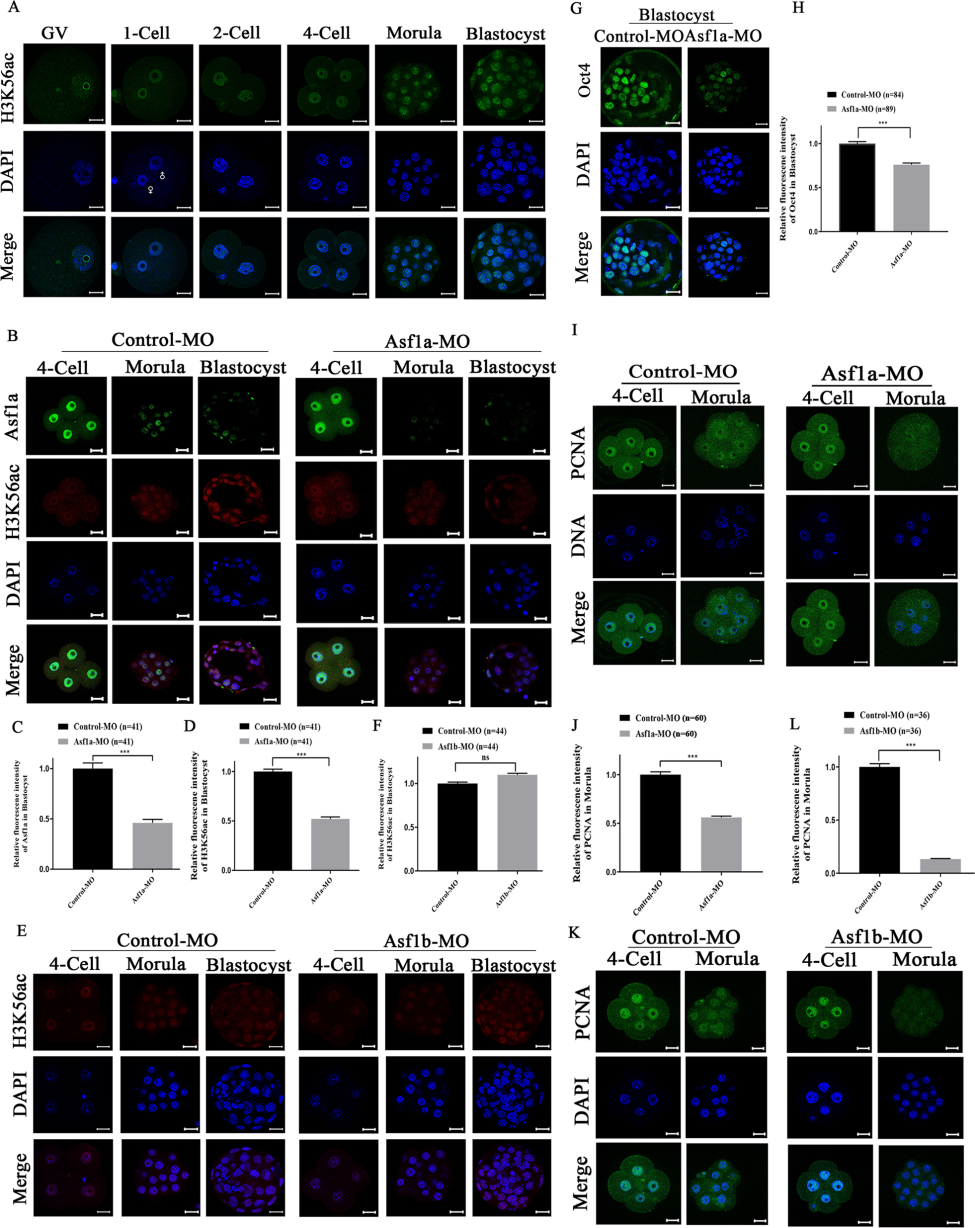
Asf1a regulates H3K56 acetylation, while Asf1b is involved in cell proliferation in early embryos. **A** Immunofluorescence detection of H3K56ac (green) in GV-stage oocytes and pre-implantation embryos. 13–45 embryos were analyzed in each group. DNA was stained with DAPI (blue). Scale bar, 20 µm. **B** Co-staining of Asf1a (green) and H3K56ac (red) in 4-cell, morula and blastocyst that were developed from Control-MO or Asf1a-MO microinjected zygotes. **C**, **D** Quantification of the fluorescence intensities for Asf1a and H3K56ac in blastocyst (Control-MO, *n* = 41; Asf1a-MO, *n* = 41). **E** Immunofluorescence detection of H3K56ac (red) in 4-cell, morula and blastocyst that was developed from Control-MO or Asf1b-MO microinjected zygotes. **F** Quantification of H3K56ac fluorescence intensity in blastocyst (Control-MO, *n* = 44; Asf1b-MO, *n* = 44). **G** Immunofluorescence detection of Oct4 in blastocyst that was developed from Control-MO or Asf1a-MO microinjected zygotes. **H** Quantification of Oct4 fluorescence intensity in blastocyst (Control-MO, *n* = 84; Asf1a-MO, *n* = 89). **I**, **K** Immunofluorescence detection of PCNA (green) in 4-cell, morula and blastocyst stage embryos that was developed from Asf1a-MO or Asf1b-MO microinjected zygotes. **J** Quantification of PCNA fluorescence intensity in morula (Control-MO, *n* = 60; Asf1a-MO, *n* = 60). **L** Quantification of PCNA fluorescence intensity in morula. (Control-MO, *n* = 36; Asf1b-MO, *n* = 36). All quantification data were presented as mean ± SEM, and analyzed using Student’s *t*-test, ***P < 0.001. DNA was stained with DAPI, scale bar 20 µm

It has been shown that the histone chaperone Asf1a in yeast and human, Asf1 in *Drosophila*, is required for H3K56ac [12, 13]. H3K56ac facilitates deposition of newly synthesized H3 molecules during DNA replication in yeast [34] and promotes embryonic stem cell pluripotency through its interaction with Oct4 in mice [35]. However, the precise role of H3K56ac in mammalian cells is not fully understood. In order to investigate the possible relationship between the histone chaperone Asf1 and H3K56ac in early embryos, we first examined the dynamics of H3K56ac in pre-implantation embryos using specific antibody. Consistent with previous studies [36], H3K56ac signal was weak but distributed throughout the GV in fully grown oocytes. After fertilization, H3K56ac was equally distributed in both female and male pronucleus in late stage zygote and present in embryonic nuclei of all analyzed developmental stages (Fig. 4A). Of note, the fluorescence intensity of H3K56ac increased gradually after fertilization and reached the highest level in blastocyst (Fig. 4A and Additional file 1: Fig. S6). Then, we microinjected Asf1a-MO in zygote and checked in the blastocyst stage embryos (Fig. 4B). Simultaneous staining of the Asf1a and H3K56ac in blastocyst stage embryos showed that fluorescence intensity of Asf1a and H3K56ac dropped to 46% (Fig. 4C) and 52% of the controls (Fig. 4D), respectively. In contrast, knockdown of histone chaperone Asf1b had no significant impact on H3K56ac levels (Fig. 4E, F). Taken together, these results indicated that Asf1a was required for H3K56ac in pre-implantation embryos.

It was reported that H3K56ac correlates positively with Oct4 binding on its target gene promoters, and their interaction promotes pluripotency both in human and mouse embryonic stem cells (ESC) [35, 37]. In order to check the possible correlation between H3K56ac and Oct4 in embryos, zygotes were microinjected with Asf1a-MO and examined at morula and blastocyst stages. We found that the fluorescence intensity of Oct4 was decreased in the Asf1a knockdown embryos at blastocyst stage (Fig. 4G, H), but not in the morula stage embryos (Additional file 1: Fig. S7A, B), indicating that H3K56ac may act in conjugation with Oct4 in blastocyst stage embryos. In contrast, Asf1b knockdown did not change fluorescence intensity of Oct4 in morula or blastocyst stage embryos (Additional file 1: Fig.S7C–F).

Previous studies have shown that Asf1b expression is abnormally high in cancer cells and closely related to cell proliferation [19–21, 38]. Thus, we asked whether Asf1b also regulates cell proliferation in early embryos. We took advantage of PCNA, which is a sliding clamp for DNA polymerase δ [39] and a universal marker of proliferating cells [40], to assess cell proliferation in early embryos. Immunofluorescence staining by specific antibody revealed that nuclear accumulation of PCNA in 4-cell was not significantly changed after Asf1b knockdown, however, PCNA was reduced to undetectable level in Asf1b-MO injected morula stage embryos (Fig. 4K). Quantification of the PCNA fluorescence intensity in morula found that nuclear accumulation of PCNA dropped to nearly 13% of the control embryos after Asf1b knockdown (Fig. 4L). Albeit mildly, Asf1a knockdown also significantly reduced nuclear accumulation of PCNA in the morula stage embryos (Fig. 4I, J). Collectively, these results indicated that both histone chaperone Asf1a and Asf1b are involved in regulation of cell proliferation during pre-implantation development, probably through different mechanisms.

## Discussion

Mammalian reproduction begins with the fusion of sperm and oocyte, followed by successive mitotic division to form a multicellular embryo. Epigenetic reprogramming is a key feature of this developmental process and nucleosome dynamic is critically required for efficient epigenetic reprogramming and normal development [41]. Since histone chaperones are actively involved in the assembly and disassembly of nucleosomes [42], we examined here the dynamics of histone chaperone Asf1a and Asf1b, and explored their potential role during fertilization and pre-implantation development in mice.

We found that nuclear enrichment of both Asf1a and Asf1b were restricted to fully grown oocyte and early cleavage stage embryos (Fig. 1A, B), which is consistent with previous reports that Asf1a and Asf1b are abundant in mature oocytes [26, 43] and highly expressed after fertilization in zygotes and 2-cell embryos [33]. Of note, observed cytoplasmic staining of Asf1b in the oocytes and embryos (Fig. 1B) might be background signal, since it was also detected after Asf1b knockdown (Fig. 3H). Interestingly, Asf1a and Asf1b have been identified as potential oocyte reprogramming factors in different mammalian species [44] and Asf1a has been demonstrated to be necessary for somatic cell reprogramming and pluripotency acquisition [45]. Therefore, it is tempting to speculate that Asf1a and Asf1b are essential maternal factors whose expressions correlate closely with totipotent or pluripotent cell status.

In contrast to *Drosophila* embryos in which Asf1 does not localize to the decondensing sperm nucleus [25], both Asf1a and Asf1b localized to the decondensing sperm shortly after fertilization in mice (Fig. 2A, B). However, our Morpholino oligos-mediated knock down (KD) experiments indicated that only Asf1a KD, but not Asf1b KD, significantly reduced H3.3 content in the paternal pronuclei (Fig. 2G, H, J, K, M and N). These findings are reminiscent of the previous reports that depletion of Asf1 from *Xenopus* egg extracts prevents de novo nucleosome assembly on prepared mouse sperm nuclei [46] and silencing Asf1 expression in *Drosophila* results in limited and insufficient paternal nucleosome assembly [25]. Hence, these evidences collectively suggest that involvement of histone chaperone Asf1 in paternal chromatin assembly is conserved in different species, which is further reinforced by recent report that de novo H3.3 deposition is dependent on Asf1 and Hira complex [47]. Since Asf1a preferentially interacts with Hira in cell extracts [9, 10, 15, 48], it is likely that interaction between Asf1a and Hira is crucial for histone H3.3 deposition on the paternal genome after fertilization. Histone H3.3 is enriched in active genes [49] and nucleosomes containing H3.3 were favorably retained at the TSSs (transcriptional start sites) in sperm chromatin [50]. However, it has been recently shown that global incorporation of H3.3 after fertilization results in relatively evenly distributed H3.3 across paternal genome in the zygotes [50], representing globally permissive chromatin environment that orchestrates ZGA (zygotic gene activation) for totipotency establishment [50–52]. Therefore, inadequate H3.3 incorporation into the paternal genome after Asf1a KD might lead to improper epigenetic reprogramming and transcriptional regulation, and eventually results in reduced developmental potential (Figs. 2G, M and 3A). Notably, decondensation of sperm nuclei was not affected by down-regulation of either Asf1a or Asf1b, indicating that histone chaperone Asf1a or Asf1b is dispensable for removal of protamine, and also confirmed our previous hypothesis that nucleosome assembly and removal of protamine in paternal pronuclei is mechanistically distinct [32].

H3K56ac is a core histone acetylation which has been indicated to play important role during early embryonic development [53]. In agreement with a previous study [36], H3K56ac is present in oocytes and all stages of pre-implantation embryos, but enriched mostly in the blastocyst stage embryos (Fig. 4A and Additional file 1: Fig. S6). Numerous studies have demonstrated that Asf1a, but not Asf1b, is required specifically for H3K56ac [11–13, 35, 54]. Consistently, we showed here in mouse embryos that while knockdown of Asf1b had little effect on H3K56ac (Fig. 4E, F), knockdown of Asf1a significantly reduced H3K56ac level, especially in morula and blastocyst stage embryos (Fig. 4B, C and D). Since H3K56ac also happens on histone H3.3 [55], the observed overall H3K56ac reduction, to some extent, might due to the insufficient incorporation of H3.3 into paternal genome after Asf1a silencing (Figs. 2G, 4B). In any case, down-regulation of Asf1a hindered pre-implantation embryonic development, at least partly through the reduced acetylation of H3K56 (Figs. 3A, 4B). H3K56ac has been shown to overlap strongly at promoters with the binding of key regulators of pluripotency, NANOG, SOX2 and OCT4, to increase their expression in human induced pluripotent cells [35, 37, 45]. Furthermore, H3K56ac interacts directly with Oct4 to promote mouse embryonic stem cell pluripotency [35]. In our study, when Asf1a expression was inhibited, the level of H3K56ac and expression of Oct4 were decreased in the inner cell mass (ICM) of blastocyst stage embryos (Fig. 4G, H), indicating that down-regulation of Asf1a leads to reduced H3K56ac, which in turn negatively impacts expression of early embryogenesis and pluripotency related genes such as Oct4 (Fig. 4B and G), and decreases developmental potentials of early embryos (Fig. 3A). Additionally, since down-regulation of H3K56ac also leads to accumulation of DNA damage [56, 57] and impairs genomic stability [33, 58], the increased DNA damage detected by γH2A.X (Additional file 1: Fig. S8) might also contribute to the significantly reduced developmental capacity (Fig. 3A, C).

Asf1b is related to meiosis onset of germ cells and loss of Asf1b reduces female reproductive capacity in mice [18]. Asf1b also plays a key role in cell proliferation and has been used as a proliferation marker, especially in cancer diagnosis [19, 20, 38]. In renal cell carcinoma, overexpression of ASF1B enhanced cell proliferation through up-regulating PCNA (proliferating cell nuclear antigen). In line with this evidence, down-regulation of Asf1b significantly reduced PCNA in the nucleus of morula stage embryos (Fig. 4K, L), indicating that Asf1b regulates developmental progression of pre-implantation embryos through fine-tuning cell proliferation [38]. Consistent with previous report in Hela cells [59], Asf1a KD also significantly reduced nuclear PCNA level in morula stage embryos (Fig. 4I, J), though much milder than that of Asf1b KD (Fig. 4L). This is not surprising given the active involvement of Asf1a and Asf1b during DNA replication, where PCNA acts as a replication clamp for DNA polymerases [60, 61]. Notably, while Asf1a preferentially interacts with Hira in vivo, Asf1b favors CAF-1 binding [15]. Since Hira is a specifc histone chaperone for histone variant H3.3 and CAF-1 escorts canonical histone H3.1/H3.2 [9, 62, 63], in the context of DNA replication, inhibition of Asf1b must impair canonical H3 deposition in the replication fork and lead to more severe replication defects than that of Asf1a KD. This is in line with our findings that Asf1b KD almost eliminated nuclear PCNA (Fig. 4L). However, it should be noted that Asf1b does not localize in the embryonic nuclei from morula stage (Figs. 1B, 3H). Therefore, how Asf1b regulates cell proliferation and embryonic development needs further investigation. Meanwhile, it will be important in the future to address at mechanistic level how the functional cross-talk between Asf1a and H3K56ac is established and fine-tunes epigenetic reprogramming and embryonic development.

## Conclusions

In this study, we found that both Asf1a and Asf1b were abundantly accumulated in 1-cell and 2-cell nuclei, but decreased or disappeared in embryonic nuclei at morula and blastocyst stages. In addition, we suggested that Asf1a, but not Asf1b, was necessary for the assembly of histone H3.3 in paternal pronuclei after fertilization in mice and the knockdown of either Asf1a or Asf1b expression compromised developmental potential of pre-implantation embryos. Moreover, we provided further evidences that reduction of H3K56ac through Asf1a KD results in decreased Oct4 expression and accumulation of DNA damage in blastocyst stage embryos. In contrast, Asf1b mainly regulates cell proliferation in early embryos.

## Materials and methods

### Collection of MII oocytes and in vitro fertilization

ICR mice were purchased from the Vital River Laboratories (Beijing, China), and maintained in a special pathogen-free facility at the Inner Mongolia University. The mice were kept in a constant light and temperature controlled environment (22–24 °C, 12 h light/dark cycle), and had free access to sufficient chow and water. Female ICR mice aged 8 weeks were superovulated by intraperitoneal injection of 10 IU pregnant mare serum gonadotropin (PMSG, Ningbo second hormone factory, Ningbo, China). 46–48 h after PMSG injection, each female mouse was injected with 10 IU human chorionic gonadotropin (HCG, Ningbo second hormone factory, Ningbo, China). Metaphase (M) II-stage oocytes were collected from the ampullae 14 h after HCG injection and treated with 1% hyaluronidase (Sigma, H3506) to remove the surrounding cumulus cells. Sperm cells were obtained from the cauda epididymis of male ICR mice and placed in HTF for capacitation. Gametes were co-incubated in HTF for 2 h for in vitro fertilization. For assessment of embryonic development, the fertilized oocytes, 2-cell, 4-cell, morula and blastocyst stage embryos were elevated morphologically at 10 hpi, 24 hpi, 48 hpi, 72 hpi and 96 hpi under a dissection microscope, and the corresponding developmental ratio was determined relative to the number of fertilized oocytes confirmed by 2^nd^ polar body extrusion and clear parental pronuclei formation. Embryos were cultured in KSOM (Sigma, 3453308) covered by mineral oil under condition of 37 °C, 5% CO_2_, and saturated humidity. All studies referring to experimental animals were performed according to the experimental protocols and standards, and approved by the Institutional Animal Care and Use Committee at the Inner Mongolia University.

### Design and microinjection of Morpholino oligos

Specific Morpholino oligos (MO) against Asf1a (Asf1a-MO), Asf1b (Asf1b-MO) and Control Morpholino (Control-MO) were designed and synthesized by Gene Tools (Oregon, USA). All Morpholino oligos were diluted with enzyme-free water to the final concentration of 1 mM and stored at room temperature. The sequences of Asf1a, Asf1b and standard Control morpholino oligos are listed as below: Asf1a-MO, 5ʹ-GTG CAT CAG CCT AGA GTA ATT CAG A-3ʹ; Asf1b-MO, 5ʹ-ATT CAG CAC CGA CAC CTT GGC CAT C- 3ʹ; Control-MO, 5ʹ-CCT CTT ACC TCA GTT ACA ATT TAT A -3ʹ. Asf1a/b-MO and Control-MO were microinjected into the cytoplasm of fertilized oocytes at 2 hpi (hour post insemination) in a droplet of M2 medium using Transfer Man 4R micromanipulator and microinjector (Eppendorf, Germany). The microinjected zygotes were cultured till blastocysts in KSOM medium (Millipore, Billerica, USA).

### Real-time quantitative PCR

20 fully grown GV oocytes or embryos collected at different developmental stages were dissolved in RNAiso Plus (Takara, Japan) and stored at – 80 °C till use. Total RNA was extracted using RNAiso Plus according to the manufacturer’s instructions. Total RNA was reversely transcribed to cDNA using PrimeScript™ RT Reagent Kit with gDNA Eraser (Takara, Japan). cDNA was used as template for quantitative PCR with TB Green ^®^Premix EX Taq TM (Takara, Japan). 12.5 μL reaction solution composed of 1 μL cDNA, 6.25 μL TB Green Premix TaqII (2×), 0.4 μL of each primer and 4.45 μL ddH_2_O was incubated in LightCycler480 real-time PCR system (Roche, Switzerland) and amplified using two-step conditions: 95 °C for 10 min; 50 cycles of 95 °C for 5 s and 60 °C for 20 s. All amplifications were done in biological triplicate with technical duplicate and the relative gene expression was normalized to embryo number (*n* = 20). Data were analyzed using LightCycler 96 SW 1.1 software and relative expression levels of the target genes were calculated by 2^−ΔΔCT^ method. The primer sequences for the Asf1a and Asf1b are listed as below: Asf1a sense: 5′-AAT GCA GGA CTC ATC CCA GAT-3ʹ; Asf1a anti-sense: 5′-TTC TTG ACC TCG GTA GGT GC-3ʹ; Asf1b sense: 5′-CTC ATT CCT GAG ACG GAC GC-3ʹ; Asf1b anti-sense: 5′-TAG CCC ACA CGG ATG AAC TC-3′.

### Immunofluorescence staining and quantification of fluorescence intensity

Oocytes and embryos were fixed in PBS containing 4% paraformaldehyde (PFA) for 20 min at room temperature, and washed three times with 1% BSA/PBS. The cells were permeabilized for 20 min using 1% BSA/PBS, 0.5% Triton X-100 and blocked for 30 min using 1% BSA/PBS, 0.1% Triton X-100. Primary antibody staining was carried out in the same buffer at 4℃ overnight. The cells were subsequently washed three times in 1% BSA/PBS (5 min each wash), incubated with secondary antibodies (Molecular Probes) for 1 h at room temperature in dark. Then, washed in 1% BSA/PBS for 5 min and stained with 4,6-diamidimo-2-phenylindole (DAPI) for 10 min. The images were taken under confocal microscopes (Zeiss). Quantification of fluorescence intensity was carried out using Image J, ROI Manager tool. First, the cytoplasmic pixel value of the protein was subtracted from the nucleus pixel value of the protein and the cytoplasmic pixel value of the DNA was subtracted from the nucleus pixel value of the DNA. Then, the ratio between the nucleus pixel value of the target protein and the nucleus pixel value of DNA was calculated as the fluorescence intensity value. The straight tool of the Image J was used to measure the diameter of male pronucleus.

### Antibodies

Primary antibodies and their dilution: Asf1a (Protein tech) 1:400; Asf1b (Protein tech) 1:400; H3K56ac (Epigentek) 1:100; H3F3B (Abnova) 1:200; Oct4 (BD Biosciences) 1:200; PCNA (Santa Cruz) 1:200. Secondary antibodies and their dilution: anti-rabbit IgG Fab2 Alexa Fluor (R) 488 Molecular Probes (Cell Signaling Technology) 1:600; DyLight 594-conjugated AffiniPure Goat Anti-Rabbit IgG (Jackson IR) 1:100; Alexa Fluor 488-AffiniPure Goat Anti-Mouse IgG (H + L)(Jackson IR) 1:200.

### Statistical analysis

Data were presented as mean ± SEM (standard error of the mean). Comparison of means was performed using the independent-samples Student’s t-test. In order to compare the differences of quantitative data between groups, normal distribution of data was verified and statistical analysis was carried out by analysis of variance (ANOVA). GraphPad Prism version 7 (GraphPad Software, La Jolla, CA, USA) was used for data analysis. *P* < 0.05 was used to determine significant difference. * indicates *P* < 0.05, ** indicates *P* < 0.01 and *** indicates *P* < 0.001.

## Supplementary Information

**Additional file 1: Figure S1.** Validation of the specificity of the Asf1a and Asf1b antibodies. (A, B) Representative IF staining of the Asf1a (A) and Asf1b (B) in the Asf1a-KD zygotes at 8 hpi. (C, D) Quantification of the fluorescence intensity of Asf1a (C, Control-MO, n=22; Asf1a-MO, n=22) and Asf1b (D, Control-MO, n=17; Asf1a-MO, n=18) in the Asf1a-KD zygotes. (E, F) Representative IF staining of the Asf1b (E) and Asf1a (F) in the Asf1b-KD zygotes at 8 hpi. (G, H) Quantification of the fluorescence intensity of Asf1b (G, Control-MO, n=23; Asf1b-MO, n=23) and Asf1a (H, Control-MO, n=11; Asf1b-MO, n=12) in the Asf1b-KD zygotes. Data were presented as mean ± SEM, and analyzed using Student’s t-test, **P < 0.01. **Figure S2.** Quantification of dynamic changes of Asf1a and Asf1b in GV oocytes and pre-implantation embryos. (A, B) Quantification of nuclear accumulation of Asf1a (A) and Aaf1b (B) in GV oocytes and pre-implantation embryos by IF stainging. Data were presented as mean ± SEM. Significant difference (a versus b, b versus c, c versus d; *P*<0.05) was determined by the one-way ANOVA and Student’s t-test. **Figure S3. **mRNA expression of the Asf1a/b in 1-cell, 2-cell, 4-cell, morula and blastocyst stage embryos derived from published RNA-seq data.** Figure S4. **Knockdown of the Asf1a/b throughout the pre-implantation embryonic development. (A, B) Representative immunofluorescence staining of the Asf1a (A) and Asf1b (B) in the pre-implantation embryos developed from zygotes injected with control or KD Morpholino oligos. (C, D) Quantification of the knockdown efficiency for Asf1a (C) and Asf1b (D) in the pre-implantation embryos. Data were presented as mean ± SEM, and analyzed using One-way ANOVA, **P < 0.01, ***P < 0.001. **Figure S5.** Nuclear localization of histone H3.3 detected by specific antibody. Fully grown GV oocytes and *in vitro* fertilized zygotes were stained with specific antibody against histone H3.3. Zygotes were collected at 2, 4, 6, 8, and 10 hpi, respectively. DNA was stained with DAPI (blue); Scale bar, 20 µm. **Figure S6.** Quantification of nuclear H3K56ac accumulation in GV oocytes and pre-implantation embryos. Data presented as mean ± SEM. Significant difference (a versus b, b versus c; *P*<0.05) was determined by the one-way ANOVA and Student's t-test. **Figure S7** Nuclear accumulation of Oct4 in the Asf1a or Asf1b knockdown embryos. (A, B) Immunofluorescence staining of Oct4 in morula (A) after knocking down Asf1a, and the quantification in morula (B, Control-MO, n=60; Asf1a-MO, n=52).(C, D) Immunofluorescence staining of Oct4 in morula (C) after knocking down Asf1b, and the quantification in morula (D, Control-MO, n=28; Asf1b-MO, n=27). (E, F) Immunofluorescence staining of Oct4 in blastocyst (E) after knocking down Asf1b, and the quantification in blastocyst (F, Control-MO, n=56; Asf1b-MO, n=29). Data were presented as mean ± SEM, and analyzed using Student’s t-test. **Figure S8.** Knockdown of Asf1a leads to increased γH2A.X in blastocyst stage embryos (A) Confocal images of γH2A.X staining at blastocyst stage. DNA was stained with DAPI (blue). Scale bar, 20 µm. (B) Quantification of the γH2A.X fluorescence intensity in blastocyst. (Control-MO, n=67; Asf1a-MO, n=67). Data were presented as mean ± SEM, and analyzed using Student’s t-test, ***P < 0.001.

## Data Availability

The data used and/or analyzed during the current study are available from the corresponding author on reasonable request.

## Abbreviations

DAPI: 4′,6-Diamidino-2-phenylindole
ESC: Embryonic stem cells
HCG: Human chorionic gonadotropin
ICM: Inner cell mass
KD: Knockdown
KSOM: potassium simplex optimized medium
MII: Metaphase II
MO: Morpholino
PFA: Paraformaldehyde
PMSG: Pregnant mare serum gonadotropin
