# Supplementary material for: Distinct role of histone chaperone Asf1a and Asf1b during fertilization and pre-implantation embryonic development in mice
Source: Epigenetics Chromatin. 2021 Dec 14;14:55. doi: 10.1186/s13072-021-00430-7 (PMC8670131; doi:10.1186/s13072-021-00430-7)
Supplement: Supplementary file 1 — Additional file 1: Figure S1. Validation of the specificity of the Asf1a and Asf1b antibodies. (A, B) Representative IF staining of the Asf1a (A) and Asf1b (B) in the Asf1a-KD zygotes at 8 hpi. (C, D) Quantification of the fluorescence intensity of Asf1a (C, Control-MO, n=22; Asf1a-MO, n=22) and Asf1b (D, Control-MO, n=17; Asf1a-MO, n=18) in the Asf1a-KD zygotes. (E, F) Representative IF staining of the Asf1b (E) and Asf1a (F) in the Asf1b-KD zygotes at 8 hpi. (G, H) Quantification of the fluorescence intensity of Asf1b (G, Control-MO, n=23; Asf1b-MO, n=23) and Asf1a (H, Control-MO, n=11; Asf1b-MO, n=12) in the Asf1b-KD zygotes. Data were presented as mean ± SEM, and analyzed using Student’s t-test, **P < 0.01. Figure S2. Quantification of dynamic changes of Asf1a and Asf1b in GV oocytes and pre-implantation embryos. (A, B) Quantification of nuclear accumulation of Asf1a (A) and Aaf1b (B) in GV oocytes and pre-implantation embryos by IF stainging. Data were presented as mean ± SEM. Significant difference (a versus b, b versus c, c versus d; P<0.05) was determined by the one-way ANOVA and Student’s t-test. Figure S3. mRNA expression of the Asf1a/b in 1-cell, 2-cell, 4-cell, morula and blastocyst stage embryos derived from published RNA-seq data. Figure S4. Knockdown of the Asf1a/b throughout the pre-implantation embryonic development. (A, B) Representative immunofluorescence staining of the Asf1a (A) and Asf1b (B) in the pre-implantation embryos developed from zygotes injected with control or KD Morpholino oligos. (C, D) Quantification of the knockdown efficiency for Asf1a (C) and Asf1b (D) in the pre-implantation embryos. Data were presented as mean ± SEM, and analyzed using One-way ANOVA, **P < 0.01, ***P < 0.001. Figure S5. Nuclear localization of histone H3.3 detected by specific antibody. Fully grown GV oocytes and in vitro fertilized zygotes were stained with specific antibody against histone H3.3. Zygotes were collected at 2, 4, 6, 8, and 10 hpi, respectively. [file 13072_2021_430_MOESM1_ESM.pdf]

Supplementary Figures

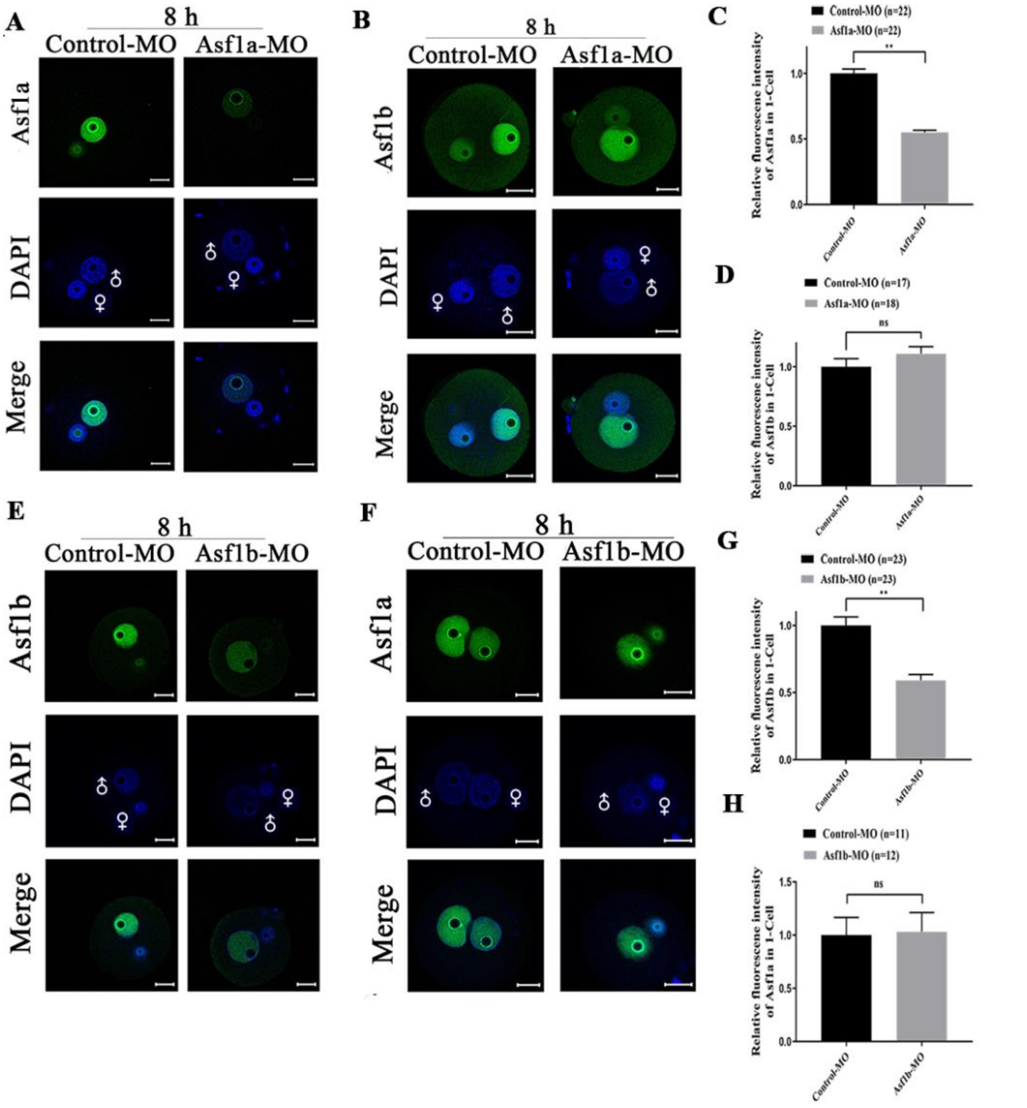

Figure S1. Validation of the specificity of the Asf1a and Asf1b antibodies.

(A, B) Representative IF staining of the Asf1a (A) and Asf1b (B) in the Asf1a-KD zygotes at 8 hpi. (C, D) Quantification of the fluorescence intensity of Asf1a (C, Control-MO, n=22; Asf1a-MO, n=22) and Asf1b (D, Control-MO, n=17; Asf1a-MO, n=18) in the Asf1a-KD zygotes. (E, F) Representative IF staining of the Asf1b (E) and Asf1a (F) in the Asf1b-KD zygotes at 8 hpi. (G, H) Quantification of the fluorescence intensity of Asf1b (G, Control-MO, n=23; Asf1b-MO, n=23) and Asf1a (H, Control-MO, n=11; Asf1b-MO, n=12) in the Asf1b-KD zygotes. Data was presented as mean ± SEM, and analyzed using Student t-test, $^{**}P < 0.01$.

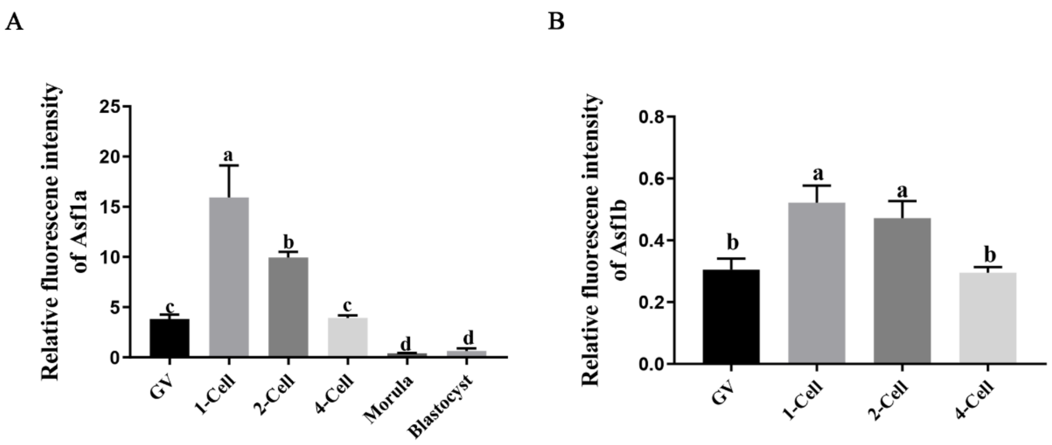

Figure S2. Quantification of dynamic changes of Asf1a and Asf1b in GV oocytes and pre-implantation embryos. (A, B) Quantification of nuclear accumulation of Asf1a (A) and Aaf1b (B) in GV oocytes and pre-implantation embryos by IF stainging. Data presented as mean ± SEM. Significant difference (a versus b, b versus c, c versus d; $P<0.05$) was determined by the one-way ANOVA and Student's t-test.

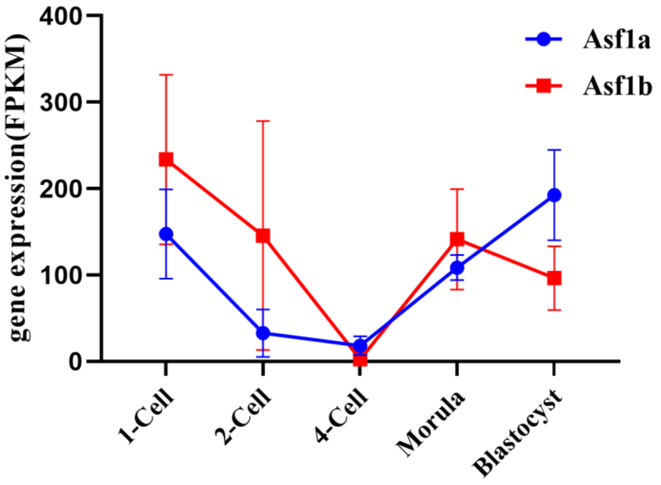

Figure S3. mRNA expression of the Asf1a/b in 1-Cell, 2-Cell, 4-Cell, Morula and Blastocyst stage embryos derived from published RNA-seq data [1].

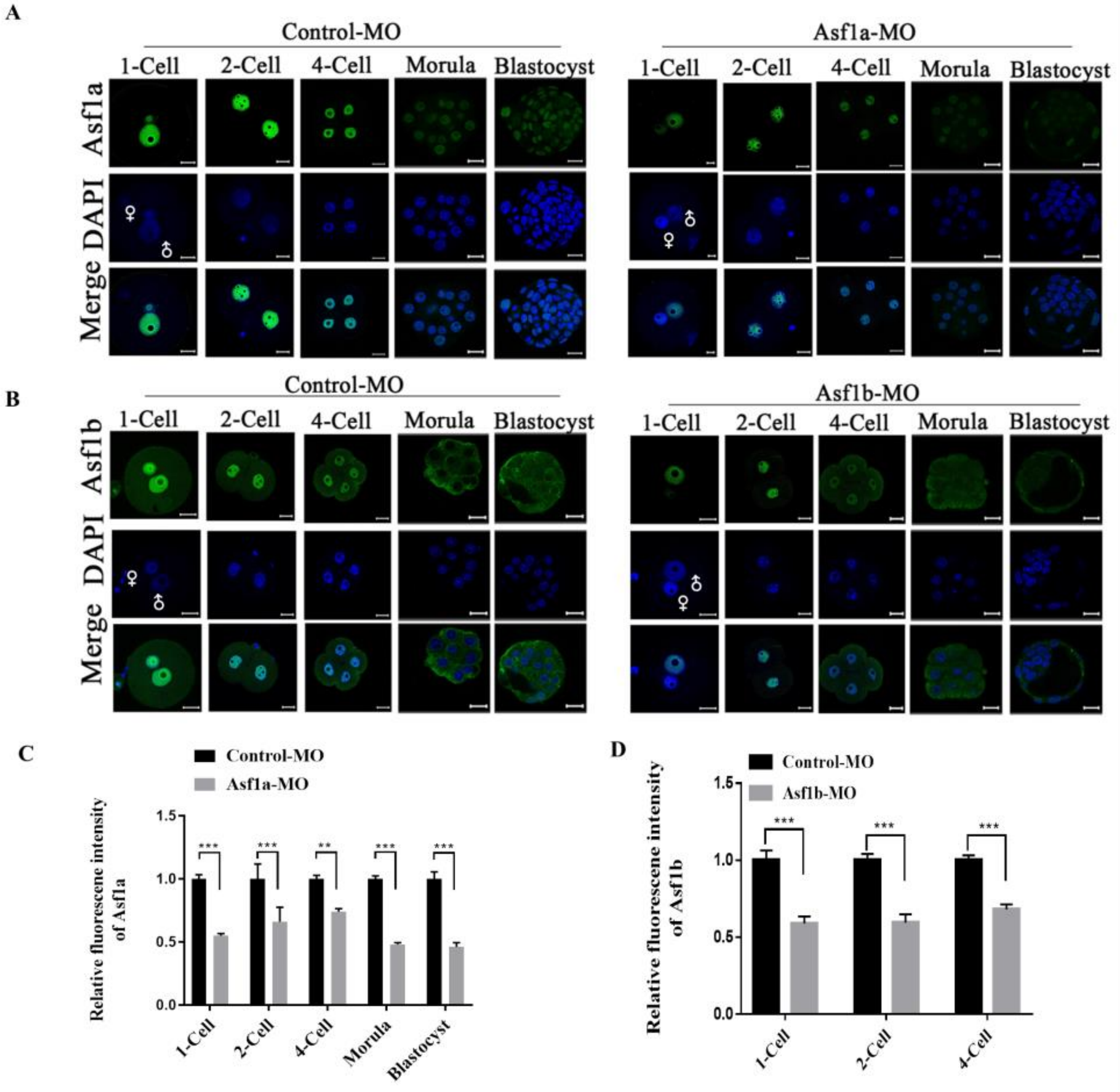

Figure S4. Knockdown of the Asf1a/b throughout the pre-implantation embryonic development. (A, B) Representative immunofluorescence staining of the Asf1a (A) and Asf1b (B) in the pre-implantation embryos developed from zygotes injected with control or KD Morpholino oligos. (C, D) Quantification of the knockdown efficiency for Asf1a (C) and Asf1b (D) in the pre-implantation embryos. Data was presented as mean ± SEM, and analyzed using One-way ANOVA, **P＜0.01,***P＜0.001.

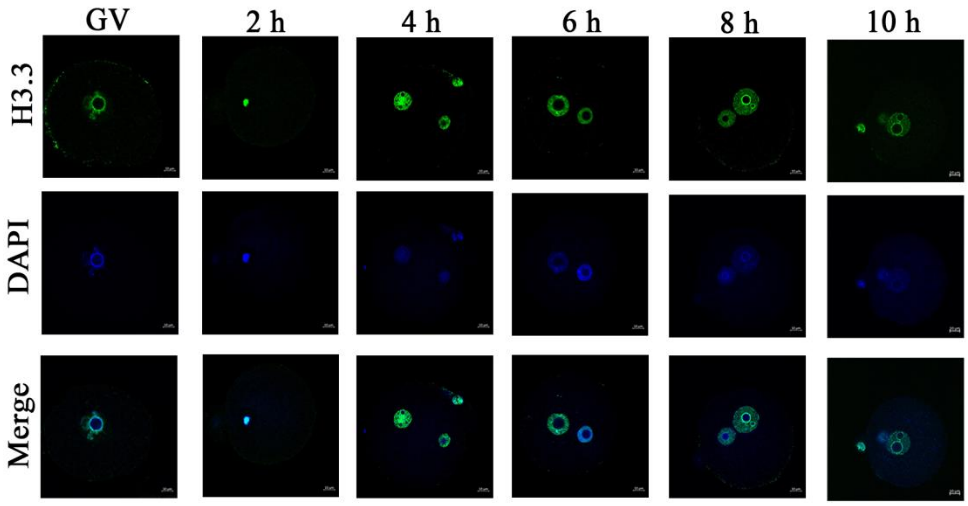

Figure S5. Nuclear localization of histone H3.3 detected by specific antibody.

Fully grown GV oocytes and *in vitro* fertilized zygotes were stained with specific antibody against histone H3.3. Zygotes were collected at 2, 4, 6, 8, and 10 hpi, respectively. DNA was stained with DAPI (blue); Scale bar, 20 μm.

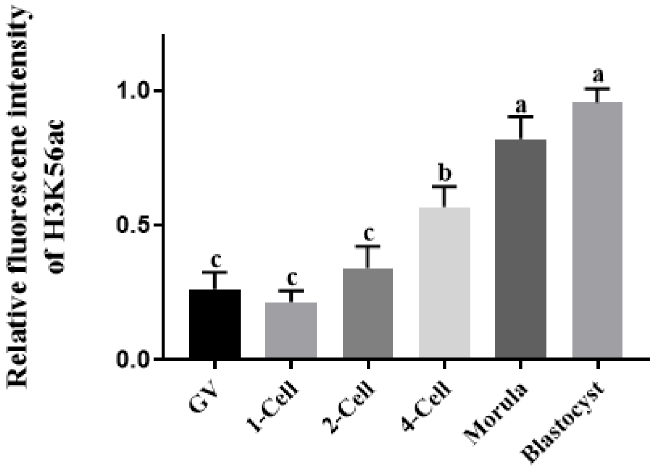

Figure S6. Quantification of nuclear H3K56ac accumulation in GV oocytes and pre-implantation embryos. Data presented as mean ± SEM. Significant difference (a versus b, b versus c; $P<0.05$) was determined by the one-way ANOVA and Student's t-test.

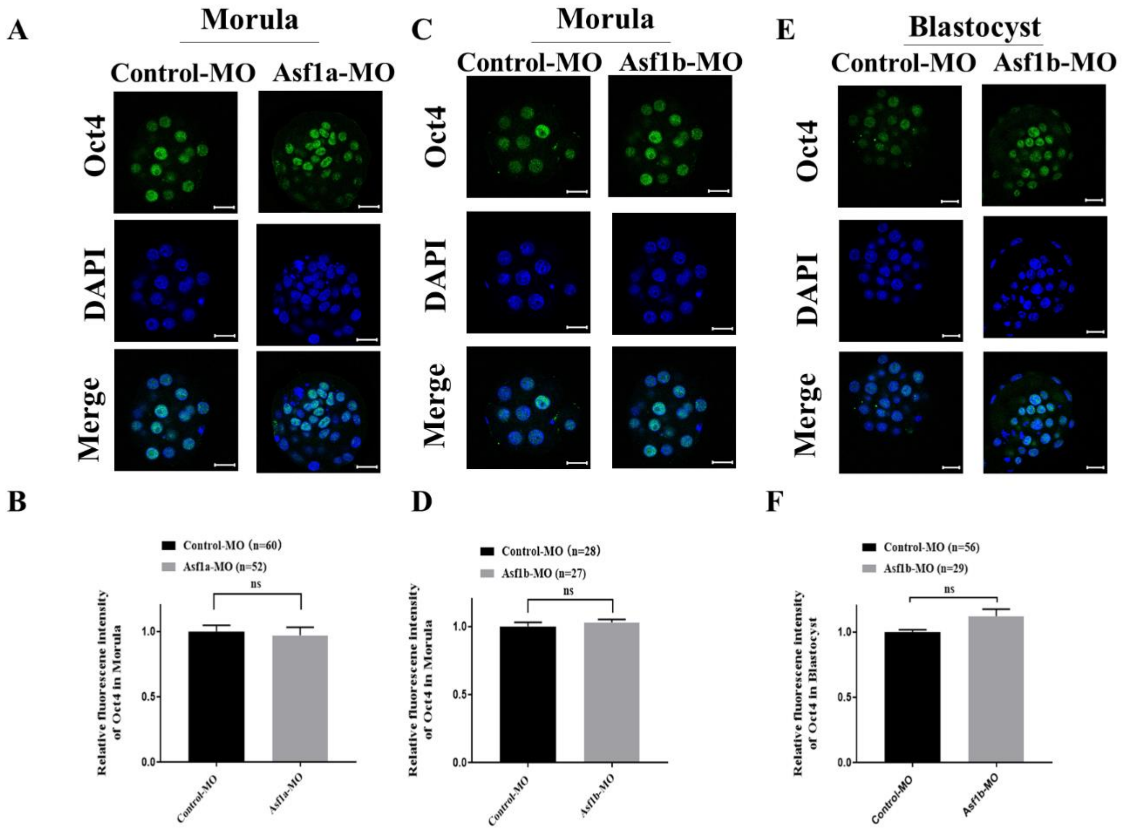

Figure S7 Nuclear accumulation of Oct4 in the Asf1a or Asf1b knockdown embryos.

(A, B) Immunofluorescence staining of Oct4 in Morula (A) after knocking down Asf1a, and the quantification in Morula (B, Control-MO, n=60; Asf1a-MO, n=52).(C, D) Immunofluorescence staining of Oct4 in Morula (C) after knocking down Asf1b, and the quantification in Morula (D, Control-MO, n=28; Asf1b-MO, n=27). (E, F) Immunofluorescence staining of Oct4 in Blastocyst (E) after knocking down Asf1b, and the quantification in Blastocyst (F, Control-MO, n=56; Asf1b-MO, n=29). Data was presented as mean ± SEM, and analyzed using Student t-test.

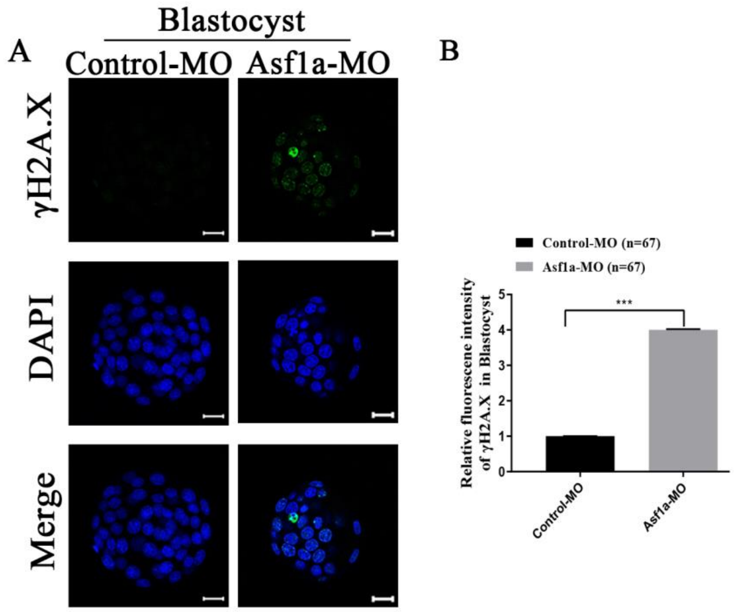

Figure S8. Knockdown of Asf1a leads to increased γH2A.X in Blastocyst stage embryos

(A) Confocal images of γH2A.X staining at Blastocyst stage. DNA was stained with DAPI (blue). Scale bar, 20 μm. (B) Quantification of the γH2A.X fluorescence intensity in Blastocyst. (Control-MO, n=67; Asf1a-MO, n=67). Data was presented as mean ± SEM, and analyzed using Student t-test, ***$P < 0.001$.

References

1. Liu W, Liu X, Wang C, Gao Y, Gao R, Kou X, Zhao Y, Li J, Wu Y, Xiu W *et al*. Identification of key factors conquering developmental arrest of somatic cell cloned embryos by combining embryo biopsy and single-cell sequencing. Cell Discov. 2016; 2:16010.
